# A Mild Catalytic Oxidation System: FePcOTf/H_2_O_2_ Applied for Cyclohexene Dihydroxylation

**DOI:** 10.3390/molecules20058429

**Published:** 2015-05-11

**Authors:** Baocheng Zhou, Wenxing Chen

**Affiliations:** 1 Department of Chemistry, Zhejiang Sci-Tech University, Hangzhou 310018, China; E-Mail: zhoubc1982@163.com; 2 Key Laboratory of Advanced Textile Materials and Manufacturing Technology, Ministry of Education of China, Zhejiang Sci-Tech University, Hangzhou 310018, China

**Keywords:** iron (III) phthalocyanine, cyclohexene, triflate, cyclohexane-1,2-diols

## Abstract

Iron (III) phthalocyanine complexes were employed for the first time as a mild and efficient Lewis acid catalyst in the selective oxidation of cyclohexene to cyclohexane-1,2-diol. It was found that the catalyst FePcOTf shown excellent conversion and moderate selectivity relative to other iron (III) phthalocyanine complexes. The optimum conditions of the oxidation reaction catalyzed by FePcOTf/H_2_O_2_ have been researched in this paper. Iron (III) phthalocyanine triflate (1 mol %) as catalyst, hydrogen peroxide as oxidant, methanol as solvent, and a mole ratio of substrate and oxidant (H_2_O_2_) of 1:1 were used for achieving moderate yields of 1,2-diols under reflux conditions after eight hours.

## 1. Introduction

In 1988, Sharpless and Jacobsen developed the OsO_4_/*N*-methylmorpholine *N*-oxide (NMO) oxidation system using cinchona alkaloid derivatives as chiral ligands for the asymmetric dihydroxylation of alkenes to obtain 1,2-diols [1]. 1,2-Diols are useful building blocks in the design of pharmaceuticals [2] and natural product synthesis [3]. These compounds can be synthesized from alkenes through alkene dihydroxylation [4]. As many alkenes can be obtained directly from petrochemicals, the conversion of alkene to 1,2-diols by oxidation is of great interest in both academia and industry. Although OsO_4_ is the most commonly used catalyst, several group VIII transition metal oxides, such as Fe [5,6,7], Ru [8,9,10], have been employed to catalyst these dihydroxylations too. The industrial use of OsO_4_ is restricted on account of its expensive price and toxic properties. Therefore, several catalysts such as polyoxometalates [11,12], sodium tungstate [13], Mg_x_Fe_3-x_O_4_ complex oxide [14], peroxovanadium [15] and oxorhenium [16] have been developed. However, most of them have limitations such as the usage of stoichiometric oxidants as well as toxic solvents and they suffer from low selectivity, harsh reaction conditions, and cumbersome work-up procedures and thus are not suitable for modern industrial practice. Therefore, it would still be highly desirable to develop new efficient methods for the dihydroxylation reaction, and clean dihydroxylations using hydrogen peroxide under mild conditions continue to be a challenge in catalysis. The selective dihydroxylation of olefins with high efficiency in terms of the amount of H_2_O_2_ is particularly difficult to achieve.

Metalloporphyrins in oxidation of substrates with various single-oxygen atom donors have played a major role in the understanding of the biologically related reactions of cytochrome P-450 [17], where oxo-metalloporphyrins are the accepted reactive intermediates. There is a stark contrast between the state of the art in porphyrin and phthalocyanine oxidation chemistry. Although phthalocyanine complexes with a similar planar structure to the porphyrins have been actively investigated as oxidation catalysts, their catalytic chemistry is practically undeveloped in terms of mechanisms and identification of the active species involved in these catalytic oxidations [18,19]. Phthalocyanines have demonstrated a wide range of applications in catalytic oxidation reactions, including alkene cyclopropanation [20], C-S bond formation [21], saturated C-H bond amination [22,23], and alkene epoxidation [24]. Our research group has been involved in the development of the catalytic chemistry of metal phthalocyanine complexes [25,26,27,28]. As for the phthalocyanine core, two methods were employed to modify the phthalocyanine. The first approach was to modify the parent phthalocyanine ring [22,29,30]. Another possible approach was to modify the parent ring system of the axial ligands, thus obtaining new complexes.

The anionic axial ligands effects of iron (III) porphyrin complexes were researched by Nam [31] and Bell [32,33,34,35]. They found that the electronegativity of anionic axial ligands affected the heterolysis or homolysis of oxoiron (IV) porphyrin intermediates, and thus decided the oxidation products. Recently Che and co-workers found that iron (III) porphyrins with triflate (CF_3_SO_3_^−^) as a counter anion show higher efficiency in the selective oxidation of terminal aryl and aliphatic alkenes to aldehydes than other counter anions, such as Cl^−^, ClO_4_^−^, and SbF_6_^−^ [36]. As for phthalocyanines, only a few cases have focused on the catalysis of iron (III) phthalocyanines [18,23]. Sorokin [37,38,39] and McGaff [40,41] reported recently that iron (III) “helmet” phthalocyanines generated by reacting iron (II) phthalocyanines with 14,28-[1, 3-diiminoisoindolinato] species can efficiently epoxidize of olefins using H_2_O_2_. It was found that the “helmet” plays an important role in the catalytic efficiency of these iron (III) phthalocyanines. White and co-workers recently developed iron (III) phthalocyanines with hexafluoroantimonate as a counter anion [FePc·SbF_6_] as catalysts showing highly selectivity in C-H amination reactions [23]. As far as we know, there is no report about the selective oxidation of cyclohexenes to 1,2-diols catalyzed by iron (III) phthalocyanines with triflate as a counter anion and using H_2_O_2_ as an oxidant.

## 2. Results and Discussion

**Scheme 1:**
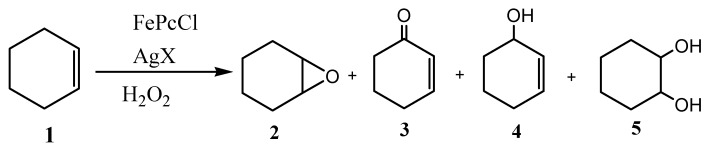
The iron (III) phthalocyanine catalyzed the oxidation of cyclohexene with H_2_O_2_.

At the outset, we examined the oxidation of cyclohexene with H_2_O_2_ using FePcX [23] (generated *in situ* by reacting commercially available FePcCl with AgX; Pc = phthalocyanine; X = NO_3_, CF_3_SO_3_, BF_4_, SbF_6_) as the catalyst. Treatment of cyclohexene with H_2_O_2_ (1.0 equiv.) in the presence of a catalytic amount of FePcX (1 mol %) in the DMF at 80 °C (Scheme 1, Table 1) did not produced detectable amounts of 1,2-diol when FePc was employed as a catalyst under these conditions (Table 1, entry 1).

**Table 1:** The selective oxidation of cyclohexene to 1,2-diols catalyzed by iron (III) phthalocyanines with different counter anions by using H_2_O_2_ as an oxidant.

Entry ^a^	Catalyst	Conversion (%)	Selectivity (%)			
			Cyclohexene oxide (2)	2-Cyclohexen-1-ol (3)	2-Cyclohexen-1-one (4)	Cyclohexane-1,2-diol (5)
1	FePc	94.0	13.7	32.4	53.9	/
2	FePcCl	85.0	59.1	16.5	21.4	3.0
3	FePcNO_3_	92.3	69.6	14.7	13.8	1.9
4	FePcOTf	97.1	20.2	18.6	15.3	45.9
5	FePcBF_4_	96.4	32.8	23.1	14.9	29.2
6	FePcSbF_6_	94.9	33.1	45.1	21.8	/
7 ^b^	AgCl	trace	ND ^c^	ND	ND	ND

^a^ Conditions: the amount of catalyst was 1 mol %; the mole ratio of substrate and oxidant (H_2_O_2_) was 1:1; reaction time was 8 h; temperature was 80 °C; solvent was DMF; ^b^ AgCl was employed in the oxidation reaction under the same conditions; ^c^ ND means not detected.

As an iron (III) porphyin with a weakly coordinating anion as the axial ligand would have the strong Lewis acidity that is needed for promoting the isomerization of epoxides to aldehydes [42], we tested the activity of five iron (III) phthalocyanines with different coordinating anions is this oxidation reaction. The conversion of cyclohexene was good to excellent in this system. Interestingly, 1,2-diol was detected in this system for the first time, which was totally different from other reports [29,30]. Replacing Cl^−^ by other anions such as NO_3_^−^, CF_3_SO_3_^−^, BF_4_^−^, SbF_6_^−^ afforded similar results (Table 1, entries 2–6). According to [42], the Lewis acidity of the metallporphyrins in the solvents used seems to affect the reactivity and selectivity of the oxidation reactions. Phthalocyanines are structurally related to porphyrins, but have a distinct framework from that of porphyrins in that all the *meso* positions of its tetrapyrrolic macrocycle system are substituted with nitrogen atoms [43]. Owing to the electron-withdrawing nature of its *meso* nitrogen atoms, the iron (III) phthalocyanine with the weaker electron donors (NO_3_^−^, OTf^−^, BF_4_^−^, SbF_6_^−^) would be a stronger Lewis acid catalyst than iron (III) phthalocyanine with the stronger electron donors (Cl^−^), and thus would be expected to exhibit enhanced reactivity and selectivity toward the oxidation reaction. Indeed, as expected, the amount of 1,2-diol increased obviously when the weaker electron donors were employed (Table 1, entries 2–6). Surprisingly, FePcOTf displayed excellent selectively to obtain 1,2-diol although NO_3_^−^ was the weakest electron donor. To explore the universality of the FePcOTf catalyst, several oxidants were tested as stoichiometric oxidants. Besides H_2_O_2_, *tert*-butylhydroperoxide (TBHP), *m*-chloroperoxybenzoic acid (*m*-CPBA) and O_2_ were also tested (Table 2). It was found that the conversion of cyclohexene was good to excellent, however the 1,2-diol was not produced using other oxidants (Table 2, entries 1,2,4). We have also optimized the reaction temperature (Table 3). As for the conversion aspect, the optimum temperature was 50 °C, but the 1,2-diol selectively rose from 17.4% to 45.9% when the reaction temperature was increased to 80 °C (Table 3, entry 3).

**Table 2:** The selective oxidation of cyclohexene to 1,2-diols catalyzed by iron (III) phthalocyanines with triflate as a counter anion and different oxidants.

Entry ^a^	Oxidant	Conversion (%)	Selectivity (%)			
			Cyclohexene oxide (2)	2-Cyclohexen-1-ol (3)	2-Cyclohexene-1-one (4)	Cyclohexane-1,2-diol (5)
1	TBHP	86.0	/	70.7	29.3	/
2	*m*-CPBA	84.0	30.0	42.1	27.9	/
3	30% H_2_O_2_	97.1	20.2	18.6	15.3	45.9
4 ^b^	O_2_	46.0	13.7	32.4	53.9	/

^a^ Conditions: the amount of catalyst was 1 mol %; the mole ratio of substrate and oxidant was 1:1; reaction time was 8 h; temperature was 80 °C; solvent was DMF; ^b^ The reaction was carried out in an autoclave under 1.5 MPa pressure.

**Table 3:** The selective oxidation of cyclohexene to 1,2-diols catalyzed by iron (III) phthalocyanines with triflate as a counter anion by using H_2_O_2_ as an oxidant at different temperatures.

Entry ^a^	Reaction temperature (°C)	Conversion (%)	Selectivity (%)			
			Cyclohexene oxide (2)	2-Cyclohexen-1-ol (3)	2-Cyclohexene-1-one (4)	Cyclohexane-1,2-diol (5)
1	25	91.0	58.2	14.4	13.4	14.0
2	50	97.0	45.3	20.3	18.0	17.4
3	80	97.1	20.2	18.6	15.3	45.9

^a^ Condition: the amount of catalyst was 1 mol %; the mole ratio of substrate and oxidant (H_2_O_2_) was 1:1; reaction time was 8 h; solvent was DMF.

In order to achieve optimum conditions, three different cyclohexene to aqueous 30% H_2_O_2_ molar ratios, *viz*. 1:1, 1:2, 1:5 were considered for a fixed amount of cyclohexene (10 mmol) and catalyst (1 mol %) in the DMF at 80 °C (Table 4). A maximum of 45.9% selectivity for the 1,2-diol was obtained for a cyclohexene to H_2_O_2_ molar ratio of 1:1 in 8 h of reaction time. The conversion was 98.0% when the ratio was 1:2 and decreased slightly at 1:5. However, cyclohexene oxide was absent when the ratio to 1:5 (Table 4, entry 3).

**Table 4:** The selective oxidation of cyclohexene to 1,2-diols catalyzed by iron (III) phthalocyanines with triflate as a counter anion using H_2_O_2_ as an oxidant at different condition.

Entry ^a^	Substrate: oxidant	Conversion (%)	Selectivity (%)			
			Cyclohexene oxide (2)	2-Cyclohexen-1-ol (3)	2-Cyclohexene-1-one (4)	Cyclohexane-1,2-diol (5)
1	1:1	97.1	20.2	18.6	15.3	45.9
2	1:2	98.0	18	30.3	12.5	39.2
3	1:5	97.2	/	30.5	37.5	32.0

^a^ Conditions: the amount of catalyst was 1 mol %; reaction time was 8 h; temperature was 80 °C; solvent was DMF.

Under the operating conditions as fixed above, the effect of catalyst considering three different amount viz. 0.1 mol %, 0.2 mol %, 1 mol %, 2 mol % as a function of time was studied and results are listed in Table 5. It is clear that 1 mol % catalyst was the best one to obtain a maximum of 97.1% conversion of cyclohexene and highest selectively for 1,2-diol (Table 5, entry 3).

**Table 5:** The selective oxidation of cyclohexene to 1,2-diols catalyzed by iron (III) phthalocyanines with triflate as a counter anion by using H_2_O_2_ as an oxidant at different condition.

Entry ^a^	Amount of catalyst (mol %)	Conversion (%)	Selectivity (%)			
			Cyclohexene oxide (2)	2-Cyclohexen-1-ol (3)	2-Cyclohexene-1-one (4)	Cyclohexane-1,2-diol (5)
1	0.1	94.2	20.0	27.4	52.6	/
2	0.5	95.0	15.2	23.1	23.2	37.5
3	1	97.1	20.2	18.6	15.3	45.9
4	2	95.5	20.0	23.2	19.4	37.4

^a^ Conditions: the mole ratio of substrate and oxidant (H_2_O_2_) was 1:1; reaction time was 8 h; temperature was 80 °C; solvent was DMF.

**Figure 1:**
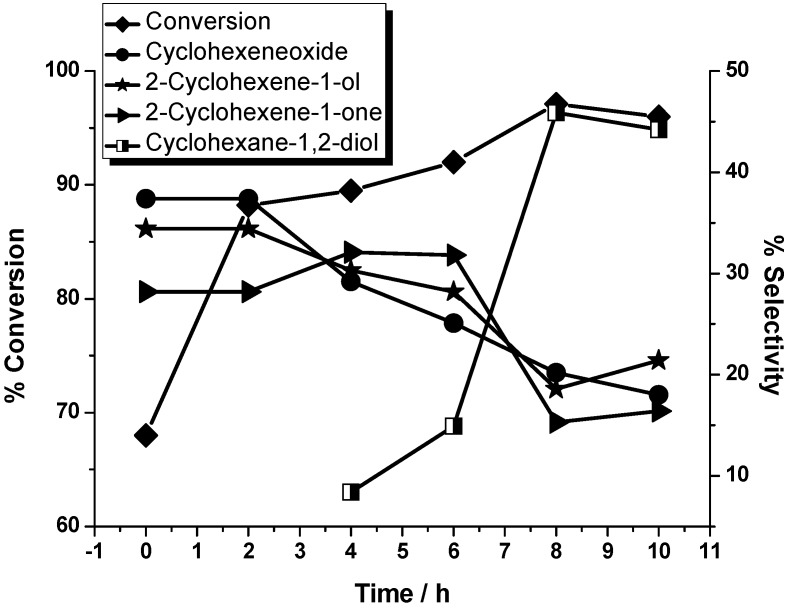
Plots showing percentage selectivity of cyclohexene oxide, 2-cyclohexene-1-one, 2-cyclohexene-1-ol and cyclohexane-1,2-diol formation and percentage conversion of cyclohexene as a function of time. Conditions: the amount of catalyst was 1 mol %; the mole ratio of substrate and oxidant (H_2_O_2_) was 1:1; temperature was 80 °C; solvent was DMF.

The conversion of cyclohexene and selectivity of the different reaction products under the optimized reaction conditions have been analyzed as a function of time and are presented in Figure 1. The time that the injection of H_2_O_2_ was finished was defined as the zero time (see the procedure in the Experimental Section). It was clear from the plot that with a conversion of 68% of cyclohexene at the zero time, no 1,2-diol was been detected. After 4 h, 8.4% selectivity of 1,2-diol has been obtained, while the conversion of cyclohexene was 89%. The selectivity of cyclohexene oxide, 2-cyclohexene-1-one, and 2-cyclohexene-1-ol obviously decreased as time goes on. After 8 h, the selectivity for 1,2-diol was the highest and reached 45.9%. At the end of 10 h, a small decrease was detected both in the conversion of cyclohexene and the selectivity of 1,2-diol.

Finally, the influence of solvent (DMF, acetonitrile, methanol) was also been researched (Table 6). 5.0% cyclohexene oxide and 52.0% 1,2-diol have been obtained when using CH_3_OH as a solvent, and the conversion of cyclohexene was a little higher than with other solvents. CH_3_OH was found to be most suitable solvent in which the iron (III) phthalocyanine complexes showed a lesser protonation tendency and sufficient stability of the formed oxo species [39].

**Table 6:** The selective oxidation of cyclohexene to 1,2-diols catalyzed by iron (III) phthalocyanines with triflate as a counter anion using H_2_O_2_ as an oxidant in different solvents.

Entry ^a^	Solvent	Conversion (%)	Selectivity (%)			
			Cyclohexene oxide (2)	2-Cyclohexen-1-ol (3)	2-Cyclohexene-1-one (4)	Cyclohexane-1,2-diol (5)
1	CH_3_OH	97.5	5.0	24.5	18.5	52.0
2	CH_3_CN	97.2	18.2	26.4	14.0	41.2
3	DMF	97.1	20.2	18.6	15.3	45.9

^a^ Conditions: the mole ratio of substrate and oxidant (H_2_O_2_) was 1:1; reaction time was 8 h; the reaction was researched under reflux condition ; the amount of solvent was 3 mL.

**Scheme 2:**
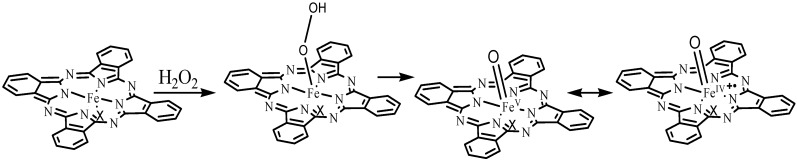
Proposed mechanism for the formation of high-valent iron oxo-phthalocyanine species.

High-valent iron oxocomplexes are well-documented and characterized by Sorokin and co-workers. High-valent iron oxocomplexes were considered be the intermediates or active species in the oxidation of olefins [39]. According to the reports, a possible mechanism for the formation of this species is proposed in Scheme 2. It should be noted that all iron (III) phthalocyanine complexes exhibit excellent conversions. However, the iron (III) phthalocyanine complexes with weaker electron donors (*i.e.*, CF_3_SO_3_^−^, NO_3_^−^, BF_4_^−^ and SbF_6_^−^) as axial ligands gave high selectivity for 1,2-diol in the H_2_O_2_ reaction, whereas the iron (III) phthalocyanine complexes with the axial ligands of stronger electron donors (*i.e.*, Cl^−^) gave no 1,2-diol in the H_2_O_2_ reaction [31]. Although we propose at this time that the electron-donating ability of the anionic axial ligands is an important factor in controlling the catalytic activity of the iron (III) phthalocyanine complexes in the H_2_O_2_ reactions, more detailed studies are in progress to gain a better understanding of the exact roles of the anionic axial ligands by density functional theory (DFT), low temperature UV-Vis and electrospray ionization mass spectrometry (ESI-MS) [37,38,39].

In summary, a mild oxidation system has been developed to convert cyclohexene into 1,2-diol, with excellent conversion. To the best of our knowledge, this is the first efficient method to prepare 1,2-diols catalyzed by the FePcOTf/H_2_O_2_ system. Such an interesting reactivity and selectivity of FePcOTf/H_2_O_2_ may provide a new strategy for preparing 1,2-diols, even regio- and stereoselective 1,2-diols. Besides, the results provide a rationale for the application of the accessible iron phthalocyanine complexes in catalytic oxidation.

## 3. Experimental Section

### 3.1. Materials

Iron (III) phthalocyanine chloride (FePcCl), AgNO_3_, AgOTf, AgBF_4_, AgSbF_6_ cyclohexene, cyclohexene oxide, 2-cyclohexen-1-ol, 2-cyclohexen-1-one, and cyclohexane-1,2-diol were purchased from Aldrich (Sigma-Aldrich Co., Ltd., Shanghai, China) and used as received. Dimethylformamide (DMF), acetonitrile, dichloromethane and methanol of gas chromatography (GC) grade were purchased from Hangzhou Jiachen Chemical Co., Ltd. (Hangzhou, China), as were *tert*-butylhydroperoxide (TBHP, 70% in water), and *m*-chloroperoxybenzoic acid (*m*-CPBA), H_2_O_2_ (9.7 M) were purchased from Hangzhou Jiachen Chemical Co., Ltd. which were used without further purification. O_2_ was provided by Hangzhou Minxing Gas Co., Ltd. (Hangzhou, China).

### 3.2. Physical Measurements

The GC traces were recorder with a GC-2014C Gas Chromatograph (Shimadzu, Suzhou, China) fitted with an FID detector, using a “Crossbond” 5% diphenyl/95% dimethylpolysiloxane capillary column (30 m length, 0.25 mm ID, 0.25 μm df) . The parameters for analysis were: injector temperature = 250 °C, detector temperature = 260 °C. Temperature program: initial temperature = 40 °C with a 2 min hold, then warmed up to 105 °C at 5 °C∙min^−1^ and held for 0 min, before finally heating up to 230 °C at 20 °C∙min^−1^. Mass spectra were recorded with a 6890N/5973I GC-MS system (Agilent Technologies Co., Ltd., Shanghai, China) coupled with an Agilent Technologies column of 30 m length, 0.25 mm internal diameter and 0.25 μm df.

### 3.3. General Procedure for the Preparation of 1,2-Diols

FePcCl (60.3 mg, 0.1 mmol) and AgX (0.12 mmol) were added to a two-necked 25 mL round-bottomed flask equipped with a condenser. DMF (3 mL) and cyclohexene (10 mmol) were added. The mixture was refluxed, then H_2_O_2_ (10 mmol) was added slowly within 30 min. The reaction mixture was allowed to cool to room temperature. The solution was diluted with CH_2_Cl_2_, and the reaction monitored over time using gas chromatography (GC). The oxidation products were identified by spiking using standards and by measurements of GC retention times in. The identity of products was also determined by a gas chromatograph connected to a mass spectrometer (GC-MS).

## 4. Conclusions

Several iron (III) phthalocyanines with different counter anions have been researched in the selective oxidation of cyclohexene using H_2_O_2_ as an oxidant. It was found iron (III) phthalocyanines with triflate as a counter anion was the best catalyst, and cyclohexane-1,2-diol was first detected in this system. A proposed mechanism for the formation of high-valent iron oxo-phthalocyanine species has been given. Besides, many effects were studied to find optimum reaction conditions. Such an amazing reactivity and selectivity of FePcOTf/H_2_O_2_ system may further develop the applied of metal phthalocyanines in catalytic oxidized organic compounds.
